# Acceptability and safety of thermal ablation to prevent cervical cancer in sub-Saharan Africa

**DOI:** 10.1186/s12885-022-09202-2

**Published:** 2022-02-02

**Authors:** Tania Metaxas, Bruno Kenfack, Jessica Sormani, Eveline Tincho, Sophie Lemoupa Makajio, Ania Wisniak, Pierre Vassilakos, Patrick Petignat

**Affiliations:** 1 Gynecology Division, Department of Pediatrics, Gynecology and Obstetrics, University Hospitals of Geneva, Boulevard de la Cluse 30, 1205 Geneva, Switzerland; 2 Faculty of Medicine and Pharmaceutical Sciences, University of Dschang, Dschang, Cameroon; 3 School of Health Sciences Geneva, HES-SO University of Applied Sciences and Arts Western Switzerland, Geneva, Switzerland; 4 Faculty of Medicine and Biomedical Sciences, University Teaching Hospital of Yaounde, Yaounde, Cameroon; 5 Geneva Foundation for Medical Education and Research, Geneva, Switzerland

**Keywords:** Cervical cancer screening, Thermal ablation, Adverse events, Sub-Saharan Africa

## Abstract

**Background:**

The World Health Organization recommends thermal ablation as an alternative to cryotherapy to treat women with precancerous lesions in low-resource settings. However, limited data are available on women’s experience and adverse events (AEs) of the procedure in the context of Sub-Saharan Africa. The objective of this study was to evaluate the acceptability and safety of thermal ablation in women screened positive for precancerous cervical lesions.

**Methods:**

Asymptomatic women aged 30–49 years old living in the Dschang Health District were invited to participate in a cervical cancer screening campaign termed “3 T-Approach” (for Test-Triage and Treat). Recruited women were asked to perform HPV self-sampling followed by triage with visual assessment and treatment with thermal ablation if required. After treatment and 4–6 weeks later, interviews were conducted to assess women’s experience on anxiety, discomfort, and pain during thermal ablation. AEs were recorded on pre-defined electronic forms 4–6 weeks after treatment to assess the procedure’s safety.

**Results:**

Between September 2018 and December 2020, 399 HPV-positive women (18.7% of women screened) were recruited, 236 (59.1%) had a positive visual assessment, 234 were treated by thermal ablation and 198 (84.6%) received therapy in the same visit. Treatment was not considered as painful (score ≤ 4/10) by 209 (90.9%) patients while 5 (2.5%) reported high pain (score 8–10/10). During post-treatment interviews 4–6 weeks later, most reported AEs were graded mild or moderate (grade I-II). The most frequent symptoms reported as mild AEs (grade 1–2) were vaginal watery discharge (75.5%), vaginal bloody-stained discharge (21.5%) and malodourous discharge (14.5%). None of the participants experienced serious AEs (grade 3–4) or AEs requiring admission to hospital or emergency consultation. The vast majority of women (99.6%) would agree to repeat the procedure if necessary and (99.6%) would recommend it to friends or family.

**Conclusion:**

Thermal ablation is widely accepted by women and appears as a safe procedure. It may contribute to improving the link between screening and treatment in a single visit and to optimizing cervical cancer control in low-resource settings.

**Trial registration:**

The study was registered on clinicaltrials.gov (NCT03757299) in November 2018 (28/11/2018).

## Background

Cervical cancer affects over half a million women worldwide every year and is responsible for more than 300′000 deaths per year, although it is a largely preventable disease through screening and treatment of precancerous lesions [1]. Cervical cancer disproportionately affects women living in low- and middle-income countries (LMICs), where nearly 90% of new cases are diagnosed [1]. However, lack of infrastructure for satisfactory implementation of vaccination and screening programs, barriers to effective treatments and lack of financial resources are key reasons of the low success of cervical cancer prevention programs in LMICs [2].

To address this gap, the World Health Organization (WHO) emphasized the importance of acting immediately to fight cervical cancer in LMICs through a comprehensive approach including three targets which should be reached by 2030: (i) vaccination of 90% of girls aged 9–14 years, (ii) screening of 70% of women with a high-performance test, and (iii) 90% of women identified with a precancerous or cancerous lesion receiving appropriate treatment and care [3].

Recent development of human papillomavirus (HPV) point-of-care assays suggests that screening women in a single visit with a high-performance test is feasible in LMIC contexts and may contribute to achieving the second target of the WHO global strategy [2]. However, to achieve the third WHO target and have an impact on the burden of disease, women screened positive for cervical precancer need to receive an effective treatment. Further, immediate treatment at the point of care is optimal in order to avoid loss to follow-up, which is of high concern in the Sub-Saharan context.

The WHO has recommended cryotherapy for the treatment of precancerous lesions, and more recently, has issued recommendation regarding the use of thermal ablation (TA) as an alternative to cryotherapy [4]. TA has been used for several decades in the United Kingdom and, in past years, has expanded to many low-resource settings where it seems to be well accepted by health care providers and patients alike [5–8]. While these results are reassuring, generalization to other sub-Saharan populations and regions should not be assumed, due to possible social and cultural differences between various settings, such as the average educational level and the population’s relationship with the health system, which may have a strong impact on the acceptability of a medical procedure such as TA.

This innovation overcomes many obstacles of cryotherapy (i.e. reduced running cost and logistical dependency on gas supply) for the treatment of women having a positive screening test [7]. In a previous cohort study conducted in 2015 including more than 1000 participants, we reported that most patients (91%) having a positive screening test were eligible for TA with a treatment success rate at 12 months of more than 70% [9, 10]. TA offers the opportunity to women living in LMICs and having a positive screening test to be treated in a single visit approach [11].

The equipment is small, portable, durable, self-sterilizing and easy to use [5, 6]. While reports about the use of TA in LMICs seems to be encouraging, there is still limited data about the quantification of pain, acceptability of the procedure and rigorous monitoring of adverse event (AEs). The aim of this study was to determine the acceptability of TA by Cameroonian women in a screen-and-treat approach and its safety profile.

## Methods

### Setting and study design

This study is nested in a larger cervical cancer screening program launched in the Dschang Health District, West Cameroon, as part of a five-year program (2018–2023). The program, termed “3 T-Approach” (for same-day test-triage and treat) combines counselling, primary HPV-based screening, visual triage and treatment of positively triaged women in a single visit. The study protocol has been described previously [12]. Briefly, after being informed about HPV infection and cervical cancer prevention, participants were invited to perform HPV self-sampling (FLOQSwabs®) using a cotton swab which was analyzed by a point-of-care HPV assay (GeneXpert®), followed by triage with visual inspection with acetic acid and Lugol’s iodine (VIA/VILI) and TA if VIA/VILI was positive. For quality control, cytology, cervical biopsies and endocervical curettage were performed for all women having a positive HPV test. Women having no lesion on visual assessment had a random biopsy at 6 o’clock at the transitional zone, while biopsies of suspected lesions were sampled when present. Sociodemographic and medical information were registered on paper case report forms and later transcribed in an online electronic database (SecuTrial®).

### Visual assessment

VIA and VILI were assessed by naked eye followed by digital imaging (native, after VIA and VILI application) captured with a smartphone (Samsung S5®) [13]. In order to optimize VIA/VILI interpretation, we used “ABCD criteria” considering as positive any cervical whitening after application of acetic acid as well as presence of spontaneous cervical bleeding; decision to treat was based on VIA/VILI assessment [14].

### Thermal ablation (TA)

Treatment was performed using a probe (WISAP; Medical Technology GmbH, Brunnthal/Hofolding, Germany) which was heated at 100° Celsius and applied on the cervix for 60 seconds after Lugol’s iodine application to delimitate the transitional zone. If necessary, the application was repeated two or more times in order to cover the entire abnormal area and transformation zone [13]. No local anesthesia was used. Women having a suspicion of cancer or lesion extending into the cervical canal which could not be covered by the probe were excluded, but treated using appropriate methods. Women were advised post-treatment to report any side effects such as abdominal pain and cramps, fever, bleeding, or vaginal discharge at the follow-up visit (4–6 weeks after treatment).

### Acceptability

Women were interviewed at the same visit after receiving treatment and 4–6 weeks later, to assess acceptability of the procedure. Respondents were invited to rate answers on a Likert scale of 1 (no acceptability) to 4 (high acceptability) [15]. Self-assessed pain was scored according to the Wong–Baker FACES® scale [16]. This validated scale consists of six different faces with a spectrum of pain intensity from 0 (no pain) to 10 (worst pain). We then formed two subgroups: normal pain (score ≤ 4), and mild pain (score >  4). An overall acceptability score based on participants’ answers using five items (anxiety, discomfort, pain, quality of information received and overall satisfaction of treatment) and attributing the same weight to each item between 0 and 10 points (maximum global acceptability of treatment) was calculated [17].

### Safety

Presence of adverse events (AEs) related to the treatment during the 30 days (4–6 weeks) after the procedure was recorded on pre-defined electronic forms. AEs were recorded and graded according to the Division of AIDS (DAIDS) Table grading the Severity of Adult Adverse Events version 2.1 [18] and the addendum 1 for the table grading female genital symptoms [18]. AEs not reported in DAIDS tables were reported as follows: Grade 1 – mild, discomfort noticed but no disruption of normal daily activity; Grade 2 – moderate – discomfort sufficient to reduce or affect daily activity; Grade 3 – severe, inability to work or perform normal daily activity; Grade 4 - life threatening, representing an immediate threat to life; and Grade 5 – death. AE severity of grade 3 and higher were considered as serious adverse events (SAEs) [17]. Healthcare providers were also questioned about their perceptions of patients’ comfort.

### Statistical analyses

Quantitative variables were expressed as means and standard deviations, and qualitative variables were expressed as percentages, unless otherwise stated. Descriptive analyses were carried out to compare women by their socio-demographic characteristics, reproductive and sexual history, disease status and other aspects. In addition, we used univariate and multivariate logistic regressions to identify socio-demographic factors associated with high scores of reported anxiety, discomfort, pain and overall acceptability. We used a two-sided level of significance of 0.05. Data were analyzed using the Stata Statistical Soft-ware Release 16 (StataCorp LP, College Station, TX, USA).

## Results

### Population and sociodemographic characteristics

Overall, 2130 women were enrolled in the “3 T-Approach” program between September 2018 and December 2020 and constitute the cohort of the present study. Among them, 399 (18.7%) were HPV-positive, and 234 (58.6%) were VIA/VILI positive and considered for TA (Table 1). Among VIA/VILI positive women, 198 (84.6%) were treated during the same day. Main reasons for treatment delay were the need for a second opinion (*n* = 19), technical problems (*n* = 6) and other reasons (menstruation, presence of cervical cysts, inability to reach the cervix). Mean age of participants was 39.2 (SD ± 6.2) years old, most of them were married or in a relationship (80.3%), and a majority completed secondary or tertiary education (68.7%). The mean age at first intercourse was 18.1 (± 2.7) years old and the median number of sexual partners was of 3 (IQR 2–5). Almost one third (30.3%) of participants had more than 5 pregnancies; and almost half (44.4%) had a desire for future pregnancy.

**Table 1:** Socio-demographic characteristics of participants (HPV-positive women, aged between 30 and 49 years old, treated with thermal ablation)

Variable	Number	Percent
Participants	234	100
Age, y mean ± SD	39.2 (± 6.2)	
Marital status		
Single/divorced/widow	46	19.7
Married/in a relationship	188	80.3
Education (*n* = 233)		
Unschooled/Primary education	73	31.3
Secondary education/University	160	68.7
Employment status		
Housewife	43	18.4
Employee/Independent/Farmer	179	76.5
Other (unemployed, student)	12	5.1
Age at menarche, y mean ± SD	14.7 (± 1.9)	
Number of sexual partners, median (IQR)	3 (2–5)	
1–5	202	86.3
> 5	32	13.7
Age at first intercourse, y mean ± SD	18.1 (± 2.7)	
≤18	152	65
> 18	82	35
Gravidity		
Nulligravida	1	0.4
1–5	120	51.3
> 5	113	48.3
Age at first delivery, y mean ± SD	21.2 (± 4.5)	
Parity		
Nulliparous	4	1.7
1–5	159	68.0
> 5	71	30.3
Having intercourse in the last 12 months		
Yes	219	93.6
No	15	6.4
Desire for future pregnancy (*n* = 232)		
Yes	103	44.4
No	129	56.6
HIV-positive (*n* = 228)		
Yes	16	7
No	212	93
Smoker		
Yes	7	3
No	227	97
HPV-Positive		
Yes	399	18.7
No	1731	81.3

Abbreviations: *N* number, *SD* standard deviation, *y* years, *HPV* human papillomavirus, *LEEP* loop electrosurgical excision procedure, *G* gravidity, *P* parity

### Acceptability

Immediately post-treatment, among the 234 women treated by TA, only 30 of them (12.8%) reported to have moderate to high anxiety and 6 of them (2.6%) felt moderate to high discomfort. Most of them (90.8%) expressed low pain scores (≤ 4) according to Wong-Baker faces, although 5 women reported a pain score of 8–10/10. The majority of women felt enough informed (97.3%), and 99.1% felt that the procedure was performed as expected or better than expected and would agree to repeat the treatment if necessary. The mean treatment’s satisfaction score was 9.9/10 (SD ± 0.8), and the Global acceptability median score was 9.1/10 (IQR 8.5–9.6) (Table 2).

**Table 2:** Acceptability at T0 (screening day)

Variable	Number	Percent
Treatment’s satisfaction^b^ (n = 232) (mean ± SD)	9.9 (± 0.8)	
Patient felt enough informed (*n* = 233)		
Yes	227	97.4
No	6	2.6
Anxiety (*n* = 231)		
No	201	87
Yes	30	13
Pain rating scale^a^ (*n* = 230) (mean ± SD)	2 (± 2)	
≤ 4	209	90.9
> 4	21	9.1
Procedure performed as expected by the patient (*n* = 231)		
Yes	229	99.1
No	2	0.9
Sufficiently informed about side effect of treatment (n = 231)		
Yes	225	97.3
No	6	2.7
Would agree to repeat treatment if necessary (*n* = 229)		
Yes	228	99.6
No	1	0.4
Would recommend screening to friends and family (n = 230)		
Yes	229	99.6
No	1	0.4
Global acceptability score^c^ (median, IQR)	9.1 (8.5–9.6)	

^a^ Pain rating scale according to Wong–Baker Faces (pain felt during the treatment, not during the biopsy) ^b ^Satisfaction scale 0 = not satisfied at all. 10 = very satisfied ^c^ Combined Anxiety, Discomfort, Pain, information received, overall satisfaction of treatment

Acceptability 4–6 weeks post-treatment showed a mean treatment acceptability score of 9.9/10 (SD ± 0.4) (*n* = 198), and a mean treatment satisfaction of 10/10 (SD ± 0.3) (*n* = 195). Ninety-nine percent (*n* = 193) of participants said they would recommend the treatment.

**Fig. 1:**
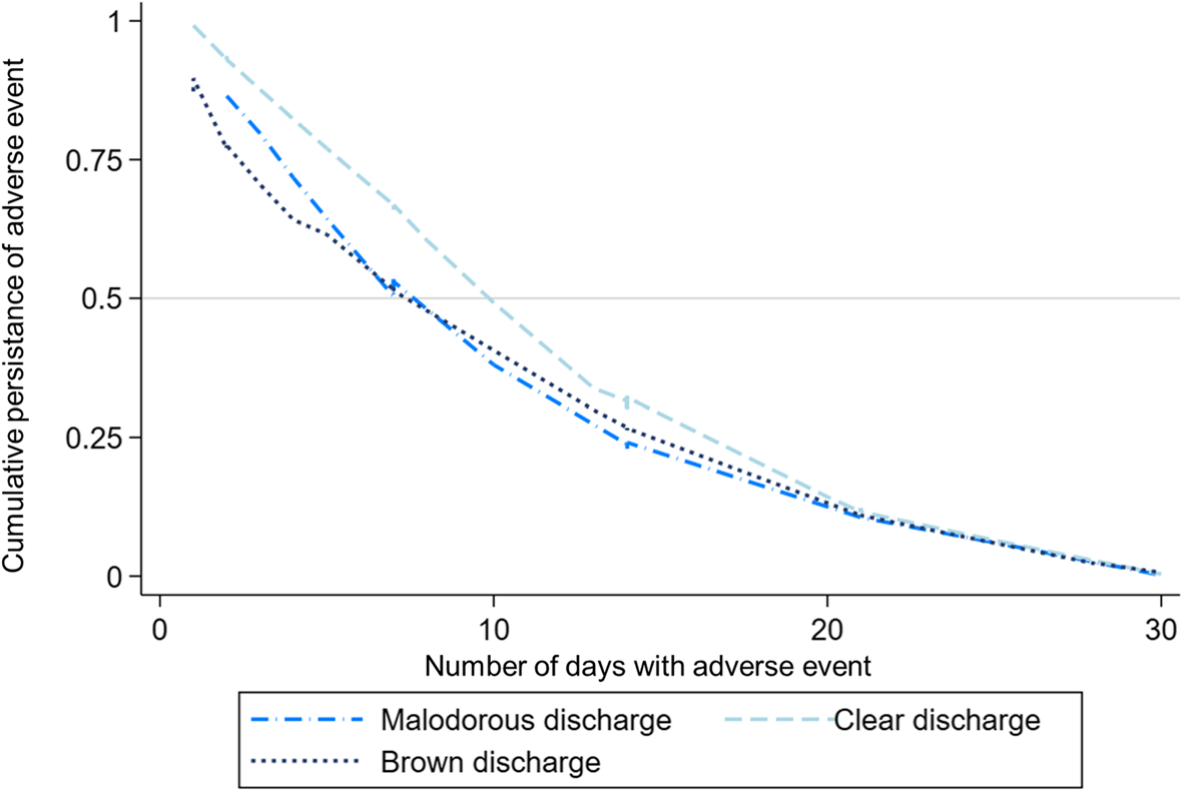
Cumulative persistence of adverse events over time following thermal ablation

*Safety* - There were no study withdrawals because of AEs (including pain during the procedure). Immediately after treatment, only few patients reported mild AEs (grade I) such as, faintness (3.4%), headache (3.1%) and nausea (0.4%). From a health care provider perspective, midwives estimated that 81.1% of patients were comfortable during the procedure (Table 3). At 4–6 weeks post-treatment, vaginal watery discharge was the most common AE graded as mild (grade I) reported by 75.8% of women, followed by vaginal bloody-stained discharge (24.2%), and vaginal malodourous discharge (14.7%). The duration of watery discharge was on average 13.1 (± 7.8) days, and 92% of women did not have it anymore after three weeks (Fig. 1). Six (2.5%) patients were prescribed topical antibiotics for infection (AE grade 2), which allowed symptom resolution, among which three (50%) were HIV-positive (Table 4). No SAEs (grade 3–4) were observed immediately after the treatment nor 4–6 weeks post-treatment. None of the participants reported any complications requiring admission to a hospital or a medical emergency room consultation.

**Table 3:** Safety Analysis after treatment at T0 (screening day)

Side effects	Number	Percent
Severity of bleeding ^a ^(*n* = 234)		
Grade 0	233	99.5
Grade 1	1	0.5
Grade 2–4	0	0
Severity of faintness (n = 233)		
Grade 0	225	96.5
Grade 1	7	3
Grade 2	1	0.5
Grade 3-4	0	0
Severity of hot flush (n = 234)		
Grade 0	230	98.5
Grade 1	4	1.5
Grade 2–4	0	0
Severity of nausea ^a^ (n = 234)		
Grade 0	233	99.5
Grade 1	1	0.5
Grade 2–4	0	0
Severity of headaches (n = 234)		
Grade 0	232	99.5
Grade 1	1	0.5
Grade 2–4	0	0
Comfortable with the treatment^b^ (n = 234)		
Yes	225	97.5
No	6	2.5

Abbreviations: *AE* adverse event, ^a^ AEs evaluated using the Division of AIDS table for grading the severity of adult and pediatric AEs, ^b^ Comfort is estimated by midwife

**Table 4:** Safety analysis after treatment at 4–6 weeks post-treatment

Side effects	Number	Percent
Watery discharge^a^ (*n* = 197)		
Grade 0	48	24.5
Grade 1	149	75.5
Grade 2–4	0	0
Days with watery discharge (*n* = 137) (mean ± SD)	13.1 ± 7.8	
Bloody-stained discharge ^b^ (*n* = 196)		
Grade 0	154	78.5
Grade 1	42	21.5
Grade 2–4	0	0
Days with bleeding (*n* = 34) (mean ± SD)	10.8 ± 8.9	
Malodorous discharge, purulent discharge ^b^ (n = 197)		
Grade 0	168	85.5
Grade 1	29	14.5
Grade 2–4	0	0
Days of malodorous/purulent discharge (*n* = 25)	10.9 ± 8.3	
Posttreatment bleeding requiring treatment (n = 196)		
Grade 0	195	99.5
Grade 1	1	0.5
Grade 2–4	0	0
Pain when urinating ^b^ (n = 196)		
Grade 0	192	98
Grade 1	4	2
Grade 2–4	0	0
Days with pain when urinating (n = 3) (mean ± SD)	4.3 ± 2.3	
Infection (n = 197)		
Grade 0	189	97
Grade 1	0	0
Grade 2	6	3
Grade 3–4	0	0
Days with infection treated with antibiotics (n = 6) (mean ± SD)	7.3 ± 4.0	
Emergency consultation n = 196		
Grade 0	196	100
Grade 1–4	0	0

^a ^Initially, the patient suffered from watery discharge, but at 4–6 weeks post-treatment she does not anymore. ^b^ AEs evaluated using the Division of AIDS table for grading the severity of adult and pediatric AEs

Univariate logistic regression showed that education was associated with anxiety, pain and with overall acceptability. Compared to women who were unschooled or with a primary education level, women having a higher education level were more likely to report higher levels of anxiety (OR, 3.35; 95%CI 1.12–9.98), higher levels of pain (OR, 10.22; 95%CI 1.34–77.72), and lower acceptability (OR, 0.22; 95%CI 0.08–0.59). Parity was also associated with anxiety and pain, with women having more than 5 children being less likely to report moderate to high anxiety (OR, 0.22; 95%CI 0.07–0.74), as well as moderate to high pain (OR, 0.16; 95%CI 0.05–0.53), compared to women with fewer children. Older women (aged > 40 years old) were less likely to experience pain than younger women (OR, 0.22; 95%CI 0.07–0.67), and had higher odds of overall acceptability (OR, 2.33; 95%CI 1.16–4.54). Finally, the desire for future pregnancy was positively associated with pain (OR, 3.49; 95%CI 1.3–9.35), and negatively associated with acceptability (OR, 0.36; 95%CI 0.18–0.7) as compared to those not wishing a future pregnancy (Table 5). When the variables were included in the multivariate logistic regression model, these results were no longer significant.

**Table 5:** Association of socio-demographic factors with anxiety, pain and overall acceptability of thermal ablation

Sociodemographic variables	Anxiety		Pain		Overall acceptability	
	OR	aOR	OR	aOR	OR	aOR
**Age**						
30–39	ref	ref	ref	ref	ref	ref
40–49	0.47 (0.21–1.05)	0.7 (0.26–1.84)	**0.22 (0.07–0.67)**	0.74 (0.29–1.9)	**2.3 (1.16–4.54)**	1.31 (0.56–3.06)
**Education**						
Unschooled/primary education	ref	ref	ref	ref	ref	ref
Secondary / tertiary	**3.35 (1.12–9.98)**	2.2 (0.68–7.16)	**10.22 (1.34–77.72)**	2.39 (0.76–7.57)	**0.22 (0.08–0.59)**	0.37 (0.13–1.06)
**Marital status**						
Single/divorced/widow	ref	–	ref	–	ref	ref
Married/in a relationship	1.67 (0.55–5.04)	–	2.48 (0.56–11.04)	–	0.34 (0.12–1.01)	0.35 (0.11–1.13)
**Employment status**						
Housewife	ref	ref	ref	–	ref	ref
Employee/Independent/Farmer	3.39 (0.77–14.93)	2.9 (0.64–13.11)	4.84 (0.63–37.25)	–	0.39 (0.13–1.17)	0.44 (0.14–1.38)
Other (unemployed, student)	6.83 (0.99–47.04)	3.18 (0.41–24.54)	3.64 (0.21–62.93)	–	**0.21 (0.04–1.00)**	0.38 (0.07–2.09)
**Number of partners**						
1–5	ref	–	ref	–	ref	ref
> 5	1.68 (0.63–4.51)	–	1.07 (0.3–3.87)	–	0.46 (0.2–1.06)	0.45 (0.18–1.14)
**Parity**						
0–1	ref	ref	ref	ref	ref	ref
2–5	0.43 (0.17–1.12)	-	**0.1 (0.03–0.31)**	-	2.03 (0.85–4.83)	1.42 (0.54–3.76)
> 5	**0.22 (0.07–0.74)**	-	**0.16 (0.05–0.53)**	-	2.48 (0.93–6.65)	1.05 (0.31–3.53)
**Desire of pregnancy**						
No	ref	ref	ref	ref	ref	ref
Yes	2.05 (0.94–4.49)	1.09 (0.42–2.85)	**3.49 (1.3–9.35)**	1.23 (0.49–3.09)	**0.36 (0.18–0.7)**	0.53 (0.23–1.24)
**Number of applications**						
1	ref	ref	ref	ref	ref	-
> 1	0.63 (0.2–1.92)	-	2.83 (0.82–9.84)	-	1.39 (0.55–3.53)	-

## Discussion

The main finding of our study is that TA appears to be highly acceptable by women, with a good global acceptability score (including anxiety, discomfort, pain, information received and overall satisfaction) (median 9.1, IQR 8.5–9.6). Almost all patients (98%) were satisfied with the treatment received, would agree to do it again if they had a recurrence of the disease, and would recommend it to a friend or family. The results are in accordance with the study of Mungo et al., conducted in a population of women living with HIV, where the vast majority also reported that they would recommend the treatment to others [9].

Pain intensity and duration as well as pain-related anxiety are factors that may influence and may serve to evaluate the effectiveness of a procedure [19]. Pain perception may differ according to the situation, previous experience of pain, trust in the health care providers as well as cultural factors. In our report, the procedure was generally well tolerated by participants, with almost 80% of them reporting none to mild pain (0–3/10) and very few of them complaining of severe pain (8–10/10). Univariate regression analysis supported that anxiety was associated with higher levels of education, higher parity and the desire for future pregnancy. However, after adjusting for multiple socio-demographic factors, no significant associations were observed. Perception of pain is important to determine if local anesthesia is required before treatment and which participants may benefit from it. In our multivariate analysis, no factors appeared to be relevant to identify which patients could benefit from anesthesia. A clinical trial conducted in Brazil addressing the question of using anesthesia prior to thermal coagulation in 100 participants, reported a significant reduction in pain [20]. However, other investigators reported essentially mild pain, while severe pain requiring hospitalization (Grade 3 or worse) was exceptional [6, 9, 12, 21, 22].

AEs grade 3 or worse associated with treatment were exceptional, and reported AEs were most of the time of grade 1 or 2 [21]. In a study conducted in Brazil, 52 women were treated without severe adverse events or complications [23]. In a screen-and-treat approach conducted in Malawi and including 381 participants, Campbell et al. reported no serious AEs in association with TA [6]. Mild vaginal discharge was experienced by most patients in our study and support that women should be advised about this symptom in the pretreatment counselling, as well as informed that it disappears after 13 days in 50% and in almost all patients (91%) after 30 days.

From a primary health care provider perspective, TA was considered as easy to perform, safe and interpreted as acceptable according to patients’ expectations and wishes. This issue is important as providers may feel more confident when patients are comfortable and satisfied [24]. In our experience, the procedure should ideally be conducted by health care providers that are well trained in pelvic examination in order to avoid any vaginal contact with the probe during the treatment process, which can be extremely painful and may cause damage to the vaginal wall. Generally, TA is a minor surgical procedure and appears to be a well-tolerated intervention by most of the patients, therefore not supporting the systematic use of local anesthesia.

This study has several limitations. First, it was conducted in a single center in a semi-rural area with few clinicians (three midwives and two part-time gynecologists) administrating screening and treatment procedures. Second, it is difficult to be sure that the anxiety, discomfort, pain and overall acceptability scores reflect only the TA treatment by itself or if women have considered the whole diagnostic and treatment process including the pelvic exam, visual assessment, cervical biopsy, endocervical brushing and TA in their responses. This potential bias has also been reported by other investigators [9, 12].

A major advantage of TA is that it may be performed immediately after visual assessment, without requiring a second pelvic exam, allowing to combine screening and treatment in a single pelvic exam and single-visit approach. This is particularly important considering the difficulty in recalling women for further management, which is a major cause of loss to follow-up and low program impact in LMICs [24] . According to the acceptability by the participants and health care providers as well as the favorable safety profile, we can expect that in the years to come, TA will become the new standard of care for women having a positive screening test in LMIC contexts [25].

## Conclusion

In conclusion, these results contribute to the evidence that TA is widely accepted by women, is safe and may become the method of choice to treat cervical precancerous lesions in low-resource settings. TA will contribute to improving feasibility of screening and treatment in a single-visit approach and optimizing programs towards elimination of cervical cancer in LMICs.

## Data Availability

The datasets used during the current study are available from the corresponding author upon reasonable request.

## Abbreviations

AEs: Adverse events
CC: Cervical Cancer
HIV: Human immunodeficiency virus
HPV: Human papillomavirus
LMICS: Low- and middle-income countries
TA: Thermal Ablation
VIA: Visual inspection with acetic acid
VILI: Visual inspection with Lugol’s iodine
WHO: World Health Organization
